# Label Free Inhibitor Screening of Hepatitis C Virus (HCV) NS5B Viral Protein Using RNA Oligonucleotide

**DOI:** 10.3390/s110706685

**Published:** 2011-06-27

**Authors:** Changhyun Roh, Sang Eun Kim, Sung-Kee Jo

**Affiliations:** 1 Radiation Research Division for Biotechnology, Advanced Radiation Technology Institute (ARTI), Korea Atomic Energy Research Institute (KAERI), 1266 Shinjeong-dong, Jeongeup-si, Jeollabuk-do 580-185, Korea; E-Mail: skjo@kaeri.re.kr; 2 Department of Nuclear Medicine, Seoul National University College of Medicine, Seoul National University Bundang Hospital, Seongnam 463-707, Korea; E-Mail: kse@snu.ac.kr

**Keywords:** viral protein, streptavidin-biotin conjugation, RNA oligonucleotide, inhibitor

## Abstract

Globally, over 170 million people (*ca.* 3% of the World’s population) are infected with the hepatitis C virus (HCV), which can cause serious liver diseases such as chronic hepatitis, evolving into subsequent health problems. Driven by the need to detect the presence of HCV, as an essential factor in diagnostic medicine, the monitoring of viral protein has been of great interest in developing simple and reliable HCV detection methods. Despite considerable advances in viral protein detection as an HCV disease marker, the current enzyme linked immunosorbent assay (ELISA) based detection methods using antibody treatment have several drawbacks. To overcome this bottleneck, an RNA aptamer become to be emerged as an antibody substitute in the application of biosensor for detection of viral protein. In this study, we demonstrated a streptavidin-biotin conjugation method, namely, the RNA aptamer sensor system that can quantify viral protein with detection level of 700 pg mL^−1^ using a biotinylated RNA oligonucleotide on an Octet optical biosensor. Also, we showed this method can be used to screen inhibitors of viral protein rapidly and simply on a biotinylated RNA oligonucleotide biosensor. Among the inhibitors screened, (−)-Epigallocatechin gallate showed high binding inhibition effect on HCV NS5B viral protein. The proposed method can be considered a real-time monitoring method for inhibitor screening of HCV viral protein and is expected to be applicable to other types of diseases.

## 1. Introduction

Hepatitis C virus (HCV) is of great interest as a worldwide infectious disease that can result in acute and chronic hepatitis, cirrhosis, and the development of hepatocellular carcinoma. Globally, approximately 3% of the world’s population or more than 170 million people are infected. HCV infection causes serious liver diseases such as chronic hepatitis, which can evolve into subsequent more serious health problems [1–4]. HCV RNA replication was catalyzed using viral polymerase nonstructural protein 5B (NS5B). This RNA-dependent RNA polymerase synthesizes a negative-strand RNA that serves as a template for the synthesis of new positive RNA strands. As a crucial and unique component of its viral replication machinery, the RNA-dependent polymerase of HCV plays a pivotal role in its life cycle [5–7]. Due to its essential role in viral replication, HCV NS5B viral protein has been mainly regarded as a prime target for antiviral therapy. For this reason, HCV NS5B is an attractive and crucial target for anti-HCV therapeutic drug discovery. Recently, the most widely analyzed method of diagnosing HCV is the detection of anti-HCV antibodies using a screening enzyme-linked immunosorbent assay (ELISA) based on recombinant proteins from the HCV genome [8]. While it is highly sensitive and specific, this assay has certain limitations [9]. For example, it cannot detect viruses during an early stage of infection, at a time when antibodies against HCV antigens are not yet being produced. In addition, the ELISA method sometimes generates false-positive or false-negative results. As another method of HCV diagnosis, the reverse transcriptase (RT)-polymerase chain reaction (PCR) method has been shown to amplify and detect HCV [10]. However, the RT-PCR method is labor-intensive, expensive, and prone to contamination. Also, the time required to perform a PCR limits its clinical application. Driven by the need to design novel approach for detection, an accurate and sensitive diagnosis of HCV diseases is crucial and essential. To overcome these bottlenecks, aptamers are introduced as an antibody and RT-PCR substitute in the application of biosensors for the detection and monitoring of biomolecules [11–14]. Aptamers are single-stranded nucleic acids that detect high affinity binding to various targets, small molecule, glycan, peptide, protein and biomolecules [15,16]. Recently, nucleic acid-based aptamers have been developed for a variety of diagnostic applications, including the detection of wide nucleic acid analytes [17]. The Octet optical biosensor platform was recently reported to be an instrument for higher-throughput, label-free, real-time molecular interaction analysis [18].

There is a need for a feasible method to detects the presence of an HCV infection, such as a direct RNA-based HCV viral protein detection method. Driven by the need to detect the presence of the HCV diseases, in this study, we demonstrate for the first time that biotinylated RNA oligonucleotide with a functional signal sequence can be used for screening and quantifying HCV RNA with selectivity and specificity. We also show that biotin-tagged RNA oligonucleotide can be used as a probe for the detection HCV viral protein NS5B and inhibitor screening on forteBio’s Octet optical biosensor system. The goal of this study is to investigate direct HCV viral protein detection and rapid inhibitor screening with a specific biotinylated RNA oligonucleotide using a streptavidin-biotin conjugation method on forteBio’s Octet optical biosensor system.

## 2. Experimental Section

### 2.1. Chemicals and RNA Oligonucleotide

(−)-Epigallocatechin gallate and cyclosporin A were purchased from Sigma-Aldrich Chemical Co. (St. Louis, MO, USA). The biotinylated group with terminal modification of NS5B RNA oligonucleotide was synthesized by BIONEER Co. Ltd. (Seoul, Republic of Korea). The biotinylated sequences of NS5B RNA oligonucleotide (NS5B: 5′-ggccacauugugaggggcuc-3′-biotin) were used as a specific probe. All other chemicals were of the highest grade.

### 2.2. Subcloning, Expression and Purification of HCV Viral Protein

The HCV NS5B gene, except for the hydrophobic C terminus 21 amino acids, was amplified using polymerase chain reaction (PCR) with a primer set, sense (5′-cgcgaattcatgtcctacacatggacagg-3′) and antisense (5′-tttctcgagtcggttggggagcaggta-3′), containing restriction enzyme sites of EcoRI/XhoI. The PCR product was digested using EcoRI/XhoI, and then ligated into EcoRI/XhoI digested expression vector pET 28a^+^ (Novagen, Madison, WI, USA), and transformed into *E. coli* DH5 α (Stratagene, La Jolla, CA, USA). The transformant was grown in a 250 mL flask containing a 50 mL Luria-Bertani (LB) medium supplemented with 50 μg mL^−1^ of kanamycin at 37 °C until the cell concentration reached an OD600nm of 0.6, and isopropylthio-β-d-galactopyranoside (IPTG) to a final concentration of 0.1 mmol L^−1^, followed by additional growth overnight at 25 °C while shaking at 180 rpm. The harvested cells were lysed using a Sonicator (W250 Sonifier, Branson, Dietzenbach, Germany). The supernatant was collected and the recombinant viral protein was purified using a Ni-nitrilotriacetic acid (Ni-NTA) affinity chromatography column (Qiagen, Germany). The supernatant was equilibrated with buffer A (10 mmol L^−1^ Tri-HCl, 500 mmol L^−1^ NaCl, 20 mmol L^−1^ imidazole, 2 mmol L^−1^ EDTA, 1 mmol L^−1^ PMSF, pH 8.0). The bound protein was then eluted with buffer B (10 mmol L^−1^ Tris-HCl, 500 mmol L^−1^ NaCl, 500 mmol L^−1^ imidazole, 2 mmol L^−1^ EDTA, 1 mmol L^−1^ PMSF, pH 8.0) at 4 °C. The purity of the purified protein was estimated using SDS-PAGE in the eluted fractions, using 10% polyacrylamide running gels [19]. The HCV NS5B viral protein was purified using a single chromatography step on a Ni^2+^ affinity column. The protein concentration was determined as described by Bradford with a bovine serum albumin (BSA) as standard [20].

### 2.3. Detection of Viral Protein NS5B and Screening of Inhibitor on Octet Platform

For the study, Octet QK, equipped with streptavidin (FA) biosensor tips, was purchased from forteBio (Menlo Park, CA, USA). Streptavidin-coated FA tips were saturated with 0.1 μg mL^−1^ biotinylated RNA oligonucleotide (15 min). The typical capture levels were 2.65 ± 0.32 nm with the standard deviation being within the instrument noise. A 10 min washing step was added. To study the interactions with biotinylated RNA oligonucleotide on the tip, a viral protein was also prepared as a five-member tenfold serial dilution with a highest concentration of 7 × 10^0^ μg mL^−1^ by 7 × 10^−4^ μg mL^−1^. Then, a viral protein of 7 × 10^0^ μg mL^−1^ to 7 × 10^−4^ μg mL^−1^ was bound to the biotinylated RNA oligonucleotide for 10 min and allowed to dissociate into a buffer (10 mmol L^−1^ potassium phosphate, 2 mmol L^−1^ EDTA, and 1 mmol L^−1^ PMSF at pH 7.4) for 10 min. Blank binding cycles containing no RNA oligonucleotide were used to correct for the baseline drift. Temperature control of the Octet was accomplished by keeping the instrument in a 22 °C temperature-controlled room so that it could be heated to 25 °C. Sensor tips were pre-wetted for 2 min in a buffer immediately prior to use, and the 96 well microplate used in the Octet was filled with 200 μL of the sample or buffer per well and agitated at 1,000 rpm. For the inhibition activity, the RNA oligonucleotide and inhibitor were facilitated through by adding on an Octet platform. The value of the signal intensity was obtained by calculating and expressing it as the mean intensity.

## 3. Results and Discussion

### 3.1. Scheme for HCV Viral Protein NS5B Detection on Octet Optical Biosensor

**Figure 1.**
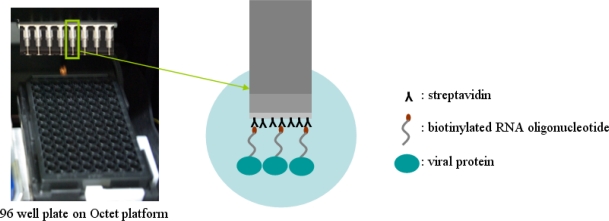
A representative scheme for detection of viral protein using streptavidin-biotin conjugation method on the Octet platform.

Specific detection of a viral protein was demonstrated using a biotinylated RNA oligonucleotide via the streptavidin-biotin conjugation method, which makes a simple analysis of RNA-protein interactions possible. The design of a sensor for effective detection of HCV viral protein using the streptavidin-biotin conjugation method is illustrated in Figure 1. The complete procedure for the detection of viral protein NS5B using the streptavidin-biotin conjugation method is as follows. First, a biotinylated RNA oligonucleotide is conjugated using a streptavidin sensor on a tip. Second, the streptavidin-biotin conjugated RNA oligonucleotide is washed to remove unspecific binding. Third, the viral protein is bound to streptavidin-biotin conjugated RNA oligonucleotide as association phase. Fourth, as dissociation phase, the biotinylated RNA oligonucleotide is analyzed in order to identify the specific detection of viral protein in the streptavidin-biotin conjugation method. To accomplish the feasibility of the targeting and monitoring, biotinylated RNA oligonucleotide conjugates having specific affinity capacity for detection of viral protein NS5B were used. Bovine serum albumin (BSA) as a reference protein was analyzed.

### 3.2. Principle of Octet Platform

**Figure 2.**
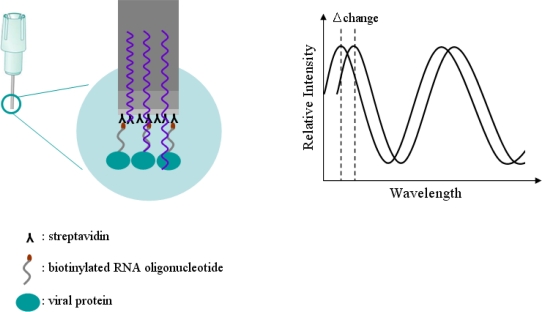
Proprietary new technology for label-free detection. A layer of molecules attached to the tip of an optic fiber creates an interference pattern at the detector with optically coated streptavidin biosensor in solution. Any change in the number of molecules bound causes a measured shift in the pattern, Biolayer Interferometry (BLI) surface chemistry. Relationship of distance between reflecting surfaces and reflected intensity would be investigated. As molecules bind, the spectrum of signal changes as a function of the layer increasing on the sensor.

Detection technology of BioLayer Interferometry (BLI) invented by Lotze [21] is a label-free, fluidics-free, real-time detection based on an optically-coated Streptavidin biosensor [22]. Interferometry is a technique based on the measurement of light intensity produced by an interference of two or more light beams. This technique can be used for detecting optical properties, such as a refraction index, and physical properties, such as the thickness of a thin film when a difference between the light beams is due to the light passing through it. The principle of the Octet optical biosensor for detection technology in the BLI method is illustrated in Figure 2.

In Figure 2, the biotinylated RNA oligonucleotide is attached to a tip coated with a streptavidin optical layer. The tip is dipped into a sample containing the target molecule. The target molecule binds to the captured molecule, and the two form a molecular layer. A white light is directed into the fiber. Two beams will be reflected to the back end. The first beam comes from the tip as a reference. The second light comes from the molecular layer. The difference in the two beams will cause a spectrum color pattern as shown here. The phase is a function of the molecular layer thickness and corresponds to the number of molecules on the tip surface. When the molecules bind to the sensor, the reflections on the internal reference layer will remain constant, while those on the interface between the molecular layer on the fiber and the solution will change with the addition of bound molecules. Bio-layer interferometry within our sensor will monitor this change. For a thin film based on an optically-coated streptavidin biosensor placed on a molecule, that is, the biotinylated RNA oligonucleotide, the two interfering beams in reflection mode can be as follows: first, a beam passing through the thin film and reflected from the interface between the substrate and the film; or alternatively, a beam reflecting from the interface between the thin film and the air. If the biotinylated RNA oligonucleotide is optically transparent, interference can also be measured in transmission mode. Interferometry can be used for detecting a change in thickness of an organic film, comprised of an RNA-protein molecules binding, consequent to exposure to a biological sample, and thereby determining the amount of RNA-protein conjugates in the sample by detecting change in thickness. Bio-layer interferometry within an Octet sensor will detect and monitor this change in real-time detection technology based on an optically-coated streptavidin biosensor. As RNA oligonucleotide binds to viral protein NS5B, the spectrum of the signal will change as a function of the signal increasing on the sensor. The Octet will monitor changes in wavelength over time. This real-time binding measurement can be used to calculate the on and off rates (Equation 1), and ultimately the concentration by plotting rates against concentration:

(1) $${\text{k}}_{\text{d}}={\text{k}}_{\text{off}}/{\text{k}}_{\text{on}}$$

We demonstrated the specific interaction between biotinylated RNA oligonucleotide and HCV viral protein on forteBio’s Octet platform, which uses biolayer interferometry to detect and quantify molecular interactions using disposable streptavidin biosensor that addresses biotinylated RNA oligonucleotide and viral protein interactions from open shaking 96 well microplates without a labeling procedure. An assay for the biotinylated RNA oligonucleotide conjugation was created on a streptavidin sensor. Streptavidin-coated FA tips were saturated with 0.1 μg mL^−1^ biotinylated NS5B RNA oligonucleotide for 900 s. The washing step allowed the removal of unspecific binding of biotinylated RNA oligonucleotide. The viral protein NS5B was prepared with a serial dilution of 7 × 10^0^ μg mL^−1^ to 7 × 10^−4^ μg mL^−1^ to study the interactions with biotinylated RNA oligonucleotide on the streptavidin sensor tip. The samples were allowed to associate for 900 s. Dissociation was measured for 600 s.

### 3.3. Expression and Purification of Viral Protein NS5B

**Figure 3.**
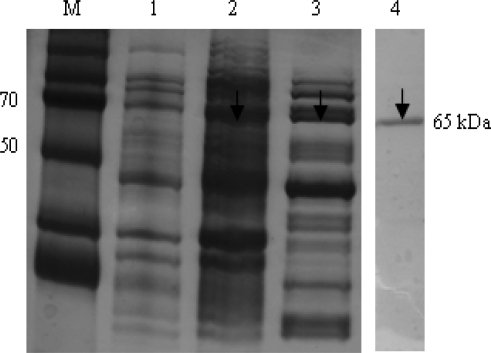
Purification of HCV NS5B viral protein. It represents the SDS-PAGE 10% gel showing NS5B protein with his-tag. M, protein marker; lane 1, before induction lysate; lane 2, total lysate; lane 3, soluble lysate; lane 4, his-tag form of purified HCV NS5B.

In Figure 3, the recombinant HCV NS5B viral protein in an *E. coli* expression system was expressed and purified using Ni-NTA affinity chromatography. In the purification step, the protein was eluted in a 1 mL fraction with a 250 mmol L^−1^ imidazole buffer. To verify the purity and homogeneity of the eluted NS5B, aliquots of eluted fractions were analyzed using Coomassie blue staining on SDS-PAGE. Eluates with the highest purity of 95% were pooled, dialyzed and stored with 50% glycerol in aliquots at −80 °C. The HCV NS5B viral protein was purified using a single chromatography step on a Ni^2+^ affinity column. The C-terminally his-tagged HCV NS5B was purified with a molecular mass of approximately 66 kDa.

### 3.4. Biosensor Effect of RNA Oligonucleotide on Viral Protein

**Figure 4.**
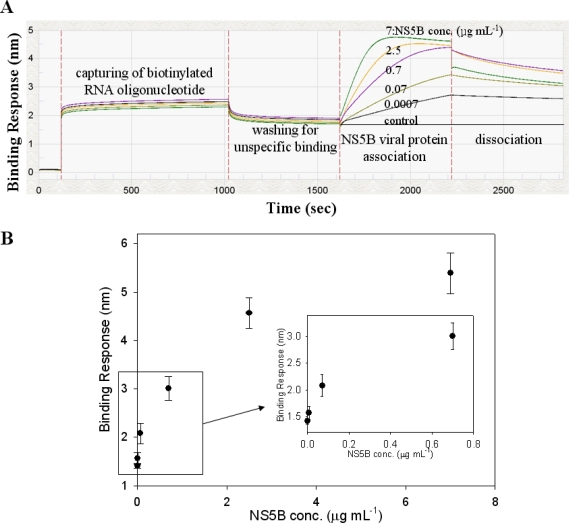
**(A)** Using the Octet interferometer (forteBio), the following assay was developed to monitor the specificity of RNA binding protein, high affinity HCV NS5B and specific RNA oligonucleotide; **(B)** Binding affinity value on streptavidin-biotin conjugation for real-time monitoring (k_d_ value).

As illustrated in Figure 4(A), the biotinylated RNA oligonucleotide showed high binding affinity signals on the streptavidin (FA) biosensor. Furthermore, Figure 4(A) shows that conjugated RNA oligonucleotide is selective for HCV NS5B viral protein at increasing protein concentrations. The binding affinity from the Octet biosensor assay with varying concentrations of the NS5B viral protein was examined, and it was observed that the signal intensity of the conjugated RNA oligonucleotide could be detected even at the lowest concentration of 700 pg mL^−1^. The signal intensity was found to increase gradually by up to 7 μg mL^−1^ of protein concentration. Figure 4A shows that the detection limit of this method was at the 700 pg mL^−1^ level. These results suggest the NS5B RNA oligonucleotide can specifically target the HCV NS5B viral protein on an Octet platform. The reason of this result will be postulated RNA oligonucleotide (5′-ggccacauugugaggggcuc-3′) used as a specific probe may interact to the active site of HCV NS5B viral protein. These results show that biotinylated RNA oligonucleotide can be effective for the quantification of HCV NS5B viral protein. In Table 1, the method and limitation for the detection of HCV viral protein were compared. The detection level of viral protein NS5B by a conventional method such as ELISA was approximately 2 ng mL^−1^ [23]. As another method, the QDs-based RNA aptamer sensor system showed a lower detection limit of 1 ng mL^−1^ for the evaluation of HCV viral protein NS5B [24]. The Octet biosensor assay using a streptavidin-biotin conjugation method showed the lowest detection limit of 700 pg mL^−1^. This result suggests that the biotinylated RNA oligonucleotide could specifically target the HCV NS5B protein in an Octet biosensor system.

In Figure 4(B), the preferential binding affinity of conjugated RNA oligonucleotide for detection of HCV NS5B viral protein was determined by analyzing the binding signal intensity measured on an Octet platform. Figure 4(B) shows that the binding affinity value was 6.3 nM. To confirm the competition reaction of specific binding, BSA was used as a negative reference. No binding affinity signal was detected with BSA because of its lack of affinity (data not shown). The signal of NS5B RNA oligonucleotide for BSA binding affinity was very similar to the background signal. Using the Octet biosensor system, we have developed a HCV detection method that can detect and quantify viral protein in solution. Here, the specific RNA oligonucleotide was examined for binding to the HCV NS5B viral protein by using a streptavidin-biotin conjugated method. It could be concluded that the detection and monitoring of viral protein by the Octet biosensor system is convenient and efficient with high specificity and sensitivity.

### 3.5. Inhibitory Effect of Viral Protein NS5B

**Figure 5.**
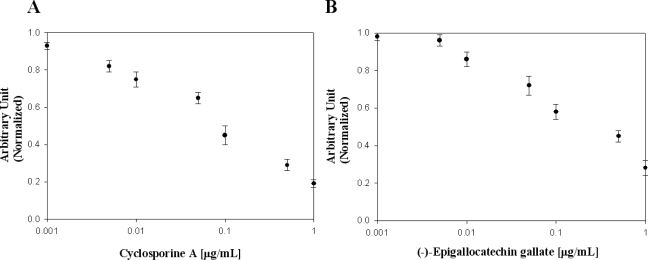
Inhibitory effect of **(A)** cyclosporin A and **(B)** (−)-epigallocatechin gallate on viral protein NS5B.

In Table 2, the effects of flavonoid compounds and cyclosporin A on the inhibition of the viral protein NS5B used in this study are presented. Many researchers have reported that cyclosporin A is one of the most remarkable drugs for viral protein NS5B. To evaluate the effects of cyclosporin A on viral protein NS5B, we used it as a known NS5B inhibitor for elucidation of our designed biochip platform [25]. We confirmed cyclosporine A concentration-dependent binding inhibition activity against viral protein NS5B [Figure 5(A)]. Among molecules screened, (−)-epigallocatechin gallate showed high binding inhibition activity [Figure 5(B)].There was no inhibition activity in flavonoids, isoflavonoids and (−)-catechin. Ivanov and Kozlov reported the inhibitory effect of trihydroxybenzaldehyde (gallic aldehyde) on NS5B protein activity [26,27]. Epigallocatechin 3-gallate, effective inhibitor such as pyrogallol derivatives was found. We demonstrated inhibitor screening on a designed Octet biosensor platform using a RNA oligonucleotide. We discovered a novel function of (−)-Epigallocatechin gallate as a binding inhibition agent on HCV NS5B viral protein. The discovery of a binding inhibition agent on HCV NS5B viral protein has been of considerable interest in developing efficient and effective methods for high-throughput screening in medicine.

## 4. Conclusions

In summary, we demonstrated a RNA aptamer system that can effectively detect and monitor HCV viral protein using biotin-based RNA oligonucleotide on an Octet platform. The monitoring of HCV viral protein has been of considerable interest in developing simple and reliable methods for HCV detection in diagnostic medicine applications. Our main goal in this study is to demonstrate the proof-of-concept that viral protein can be detected and monitored with remarkable selectivity and specificity. For application, this designed platform for a novel inhibitor assay possesses significant potential as a target screening. As these results show, RNA-protein interactions may be of great utility in the identification of novel RNA binding proteins and in understanding how proteins recognize particular RNA sequences. An RNA oligonucleotide biosensor based on the streptavidin-biotin conjugation method has attracted significant attention and has become a promising method for the specific detection of oligonucleotide because of its high sensitivity, low cost, rapid response, and low labor-intensive requirements. The proposed method can be considered an efficient strategy and new promising platform for monitoring applications.

## Figures and Tables

**Table 1.** The method and limitation for detection of HCV viral protein.

**Source**	**Method**	**Probe**	**Detection limit**	**Reference**
NS5B	ELISA	antibody	2 ng mL^−1^	22
NS5B	Biochip	QD-based RNA Oligonucleotide	1 ng mL^−1^	23
NS5B	Octet biosensor	biotinylated RNA Oligonucleotide	700 pg mL^−1^	In this study

**Table 2.** The effects of flavonoid compounds and cyclosporine A on the binding inhibition of viral protein NS5B used in this study.

**Compounds**	**Inhibition Effect**
Naringenin	−
Hesperetin	−
Apigenin	−
Luteolin	−
Quercetin	−
Rutin	−
Fisetin	−
Myricetin	−
(−)-Catechin	−
(−)-Epigallocatechin 3-gallate	+
Biochanin A	−
Formononetin	−
Daidzein	−
Genistein	−
Glycitein	−
Cyclosporin A	+

+: It means this compound has inhibition activity on viral protein NS5B.

−: It means this compound has no inhibition activity on viral protein NS5B.
